# The Alboran volcanic-arc modulated the Messinian faunal exchange and salinity crisis

**DOI:** 10.1038/s41598-018-31307-7

**Published:** 2018-08-29

**Authors:** Guillermo Booth-Rea, César R. Ranero, Ingo Grevemeyer

**Affiliations:** 1 Instituto Andaluz de Ciencias de la Tierra (UGR-CSIC), Granada, Spain; 2 Department of Geodynamics, University of Granada, Granada, Spain; 3 Barcelona Center for Subsurface Imaging, Instituto de Ciencias del Mar, CSIC, Pg. Marítim de la Barceloneta 37-49, 08003 Barcelona, Spain; 4 ICREA, Pg. Lluís Companys 23, 08010 Barcelona, Spain; 5 GEOMAR, Helmholtz Centre for Ocean Research Kiel, Kiel, Germany

## Abstract

What process triggered the Mediterranean Sea restriction remains debated since the discovery of the Messinian Salinity Crisis (MSC). Recent hypotheses infer that the MSC initiated after the closure of the Atlantic-Mediterranean Betic and Rifean corridors, being modulated through restriction at the Gibraltar Strait. These hypotheses however, do not integrate contemporaneous speciation patterns of the faunal exchange between Iberia and Africa and several geological features like the evaporite distribution. Exchange of terrestrial biota occurred before, during and after the MSC, and speciation models support an exchange path across the East Alborán basin (EAB) located a few hundreds of km east of the Gibraltar Strait. Yet, a structure explaining jointly geological and biological observations has remained undiscovered. We present new seismic data showing the velocity structure of a well-differentiated 14–17-km thick volcanic arc in the EAB. Isostatic considerations support that the arc-crust buoyancy created an archipelago leading to a filter bridge across the EAB. Sub-aerial erosional unconformities and onlap relationships support that the arc was active between ~10–6 Ma. Progressive arc build-up leading to an archipelago and its later subsidence can explain the extended exchange of terrestrial biota between Iberia and Africa (~7–3 Ma), and agrees with patterns of biota speciation and terrestrial fossil distribution before the MSC (10–6.2 Ma). In this scenario, the West Alboran Basin (WAB) could then be the long-postulated open-marine refuge for the Mediterranean taxa that repopulated the Mediterranean after the MSC, connected to the deep restricted Mediterranean basin through a sill at the Alboran volcanic arc archipelago.

## Introduction

**Figure 1:**
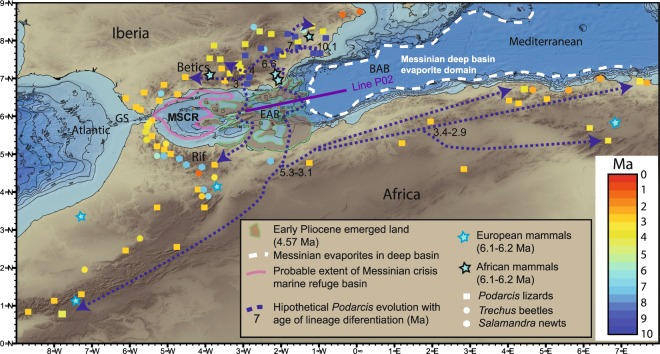
Faunal exchange patterns between Africa and Iberia from phylogenetic studies^13,15,16^ and the fossil record^17–19^. Main phylogenetic mitochondrial-DNA lineage splits of *Podarcis* lizards^13^, *Trechus* beetles^15^ and *Salamandra* newts^16^ are age color coded. Also shown is the location of the late Miocene Alborán volcanic arc archipelago in the East Alborán Basin (EAB) inferred from the extent of emerged land during the Early Pliocene, marked by the brown-filled green poligons^27^. The presently submerged volcanic arc is crossed by wide-angle seismic line P02 analyzed in this work. Notice the extent of Messinian deep-basin salt deposits bounded by a dashed white line in the Balearic-Algerian Basin (BAB). A pink line in the western Alborán basin maps the probable extent of a Messinian Salinity Crisis Refuge (MSCR) to the east of the Gibraltar Strait (GS). Blue dashed line and arrows indicate the patterns of exchange of *Podarcis* lizards between Africa and Iberia, and their diversification and speciation through time, coded by a color scale in Ma. The figure was done with GMT 5 (General Mapping Tools, (https://www.soest.hawaii.edu/gmt/) and modified further with Adobe Illustrator CS5 (https://creative.adobe.com/es/products/download/illustrator).

Models for the onset of the MSC^1–3^ infer that uplift (perhaps related to lithospheric delamination) closed the Betic and Rifian marine gateways^4,5^ at ~7.6 Ma^6^ and ~6.7–6.2 Ma^3^ respectively (Fig. 1), which triggered evaporite deposition in the Mediterranean starting at 5.97 Ma^7^. Implicitly assumed in most models is that the Gibraltar marine gateway was closed before^1^, and that the MSC ended when the straits were breached^8^ at ~5.3 Ma^2^. However, recent work suggests that the Strait of Gibraltar was always open as a watergate to the MSC^3,9^, and that the Mediterranean was probably a deep basin during the MSC^9–11^. Thus, requiring an alternative land bridge to the Gibraltar Strait for terrestrial-fauna exchange between Africa and Iberia.

During the MSC, Africa and Iberia exchanged terrestrial fauna^12^, but mitochondrial DNA phylogenetic studies provide evidence of exchanges that are both older^13–16^ and younger, requiring longer-lasting temporal land bridges^12^ (Fig. 1). During part of the Tortonian (10–7.2 Ma) when great part of southern Iberia was below sea level^17^, taxa speciation patterns indicate that SE Iberia was an important center of diversification before the MSC^13^ requiring local emerged biological hotspots (Fig. 1). First documented speciation initiated in SE Iberia between 10–8 Ma, with *Podarcis* and *Timon* lizards^13,14^ that irradiated towards the western Betics and Rif since the late Miocene (~7 Ma). For instance, some African *Podarcis* lizards separated from a common Iberian ancestor at ~6.6 Ma and after the MSC radiated towards the Atlas, Algeria and Tunisia^13^ differentiating into lineages (Fig. 1). Ground beetles of the *Trechus fulvus* group irradiated twice from SE Iberia towards the eastern Rif, at ~6.9 and ~6.3 Ma^15^. Post-MSC diversification (~5–3 Ma) of exchanged taxa occurred from the eastern Rif towards the western Rif and Algeria for *Podarcis* lizards^13^, *Salamandra* newts^16^ and *Trechus* beetles^15^ (Fig. 1). Moreover, exchange and speciation from Iberia towards the Rif still continued at ~3 Ma for the *Podarcis voucheri* lizard lineage^13^.

The large terrestrial vertebrate fossil record also supports that faunal exchange between Africa and Iberia occurred before the MSC^17–19^. The first Late Miocene large vertebrate exchange between Africa and Iberia is dated at ~6.2 Ma, or slightly before for *Hippopotamou*s/*Hexaprotodon crusafonti*^19^, being recorded in the eastern basins of the Betics and Rif^17–19^ (Fig. 1). In this scenario, the Gibraltar Strait is interpreted as an effective physical barrier to genetic exchange during the Messinian time^12^, with controversial evidence of terrestrial biota exchange occurring only during the MSC^12–16^. In accord, the Gibraltar Strait region was the last to be colonized in late Pliocene times by most exchanged taxa^13–16^ (Fig. 1).

It is well established that the closure of the Betic-Rif marine gateways predates the MSC^3,9^, requiring an additional sill to modulate water inflow from the Atlantic Ocean to trigger the MSC. Work in the Gulf of Cadiz, west of the Gibraltar Strait, supports that the main growth phase of the accretionary prism occurred before the MSC and no clear tectonic pulse has been identified yet for the hypothesized closure of the Gibraltar Strait^20^. The proposed Zanclean catastrophic flooding of the basin with Atlantic water, forming subaerial canyons later filled by Pliocene sediments^8,21^, requires a closure of the Gibraltar Strait and desiccation of the WAB during the deep halite-phase of the MSC. This desiccation phase is supported onshore by canyons filled by Zanclean sediments in the Betics and Rif ^22,23^. However, complete desiccation in the deeper basin is at odds with the requirement of continuous connectivity between the Atlantic and Mediterranean during the MSC^3,9–11,24^. The top of the Messinian unit is indeed deeply incised near the Gibraltar Strait in the WAB, although recent work suggests that the canyons at the Gibraltar Strait may be older than Zanclean, developed during the MSC and under submarine conditions^9^_._

A permanent submarine environment in the WAB agrees with models that seek to explain the volume of salt deposited proposing a two-way connectivity between the Atlantic and the Mediterranean during the first (5.96 to 5.6 Ma) and later stage (5.5 to 5.3 Ma) of the MSC^3^. The coexistence of an open-marine WAB with a deep restricted Mediterranean^9–11^ during the phase of maximum restriction (5.6–5.5 Ma) could be solved with a sill East of the WAB.

**Figure 2:**
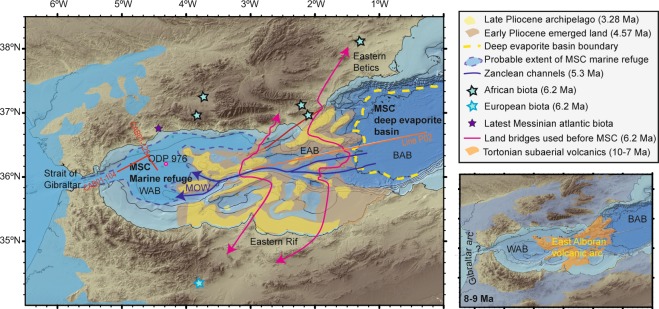
(**a**) Map of the western Mediterranean during the MSC, including features from before and after the desiccation event. Archipelago dimensions are inferred from erosional unconformities mapped with seismic data^27^. Notice that together the distribution of unconformities and transgressive sediment onlap over the volcanic basement^27^ support the progressive submergence of the volcanic archipelago between the Early and Late Pliocene. In this scenario, 5.3 Ma Zanclean channels could result from dense Mediterranean Out Flow Waters (MOW). ODP 976 core shows unrestricted marine depositional environment for the Messinian-Pliocene sequence^26^ (although the uppermost Messinian is missing from erosion) supporting a marine refuge in the Western Alboran Basin during the MSC. (**b**) Paleogeographic reconstruction of the westernmost Mediterranean Alboran volcanic archipelago during the Tortonian, around 8–9 Ma. Non-volcanic islands in the Betics and Rif included in the figure according to Gibert *et al*.^17^. Areas in dark orange where probably subaerial volcanoes showing no pre-MSC sedimentary cover. The figure was done with GMT 5 (General Mapping Tools, (https://www.soest.hawaii.edu/gmt/) and modified further with Adobe Illustrator CS5 (https://creative.adobe.com/es/products/download/illustrator).

A volcanic arc crops out onshore along Cabo de Gata and the eastern Rif coast, and extends offshore across the EAB (Fig. 2), where dredging of some basement outcrops recovered late Miocene island arc lavas^4,5^. But interpretation of these rock samples is debated, and while geochemical arguments indicate a developed volcanic arc^4,5^, and seismic images support it^25^, other studies infer that rock samples represent local intrusions of otherwise little modified continental crust^26,27^.

The nature of the crystalline crust of the EAB is inadequately known partially due to the lack of modern deep-penetrating wide-angle seismic data. We have collected and modeled wide-angle seismic lines in the EAB and the Balearic-Algerian Basin (BAB), with line P02 co-located with an existing deep-seismic reflection image^25^ extending for ~250 km from the back-arc to the arc (Figs 1 and 2). We have reinterpreted two industry seismic reflection lines to analyze the nature and geometry of the Messinian-Pliocene transition in the WAB. We have applied basic isostatic considerations to the resulting crustal structure to evaluate the possibility of an emerged domain across the EAB. Finally, we discuss our new findings under the light of other geological data and previously published faunal taxa speciation patterns.

## Methods

### Seismic experiment and data

**Figure 3:**
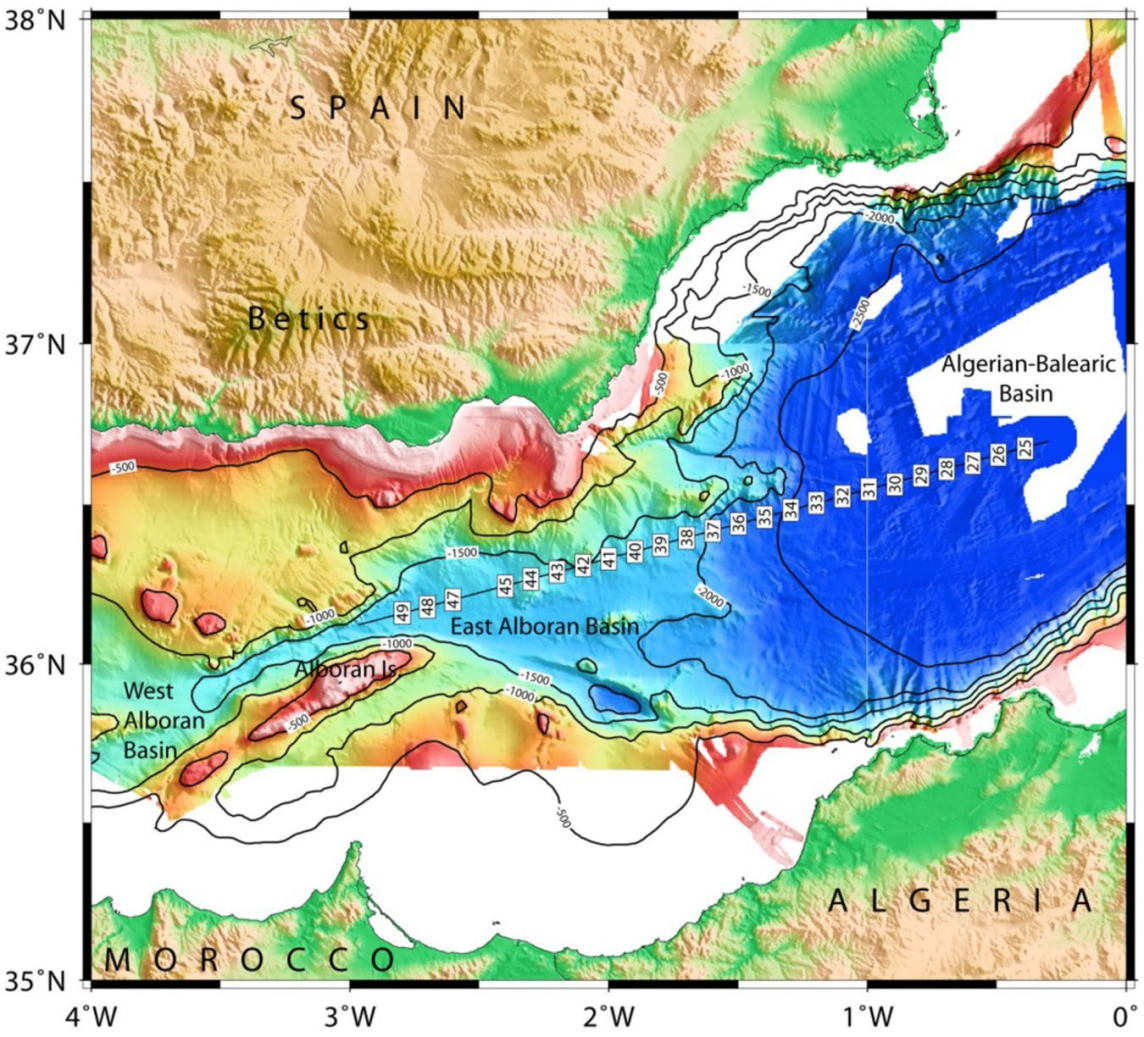
Location map of line P02 running from the Algerian-Balearic Basin into the East Alborán Basin. The figure was done with GMT 5 (General Mapping Tools, (https://www.soest.hawaii.edu/gmt/).

During the WESTMED experiment in 2006, we obtained seismic refraction and wide-angle data in the Alborán Sea and the Algerian-Balearic Basin with the German research vessel METEOR. Profile P02 was shot along the previously analyzed multi-channel-seismic MCS profile ESCI-Alb 2^25^, running from longitude 0.4°W to 3°W, covering the western part of the Algerian-Balearic basin and the East Alborán basin, terminating to the north of Alborán Island (Fig. 3). In total 25 Ocean-Bottom-Seismometers (OBS) and Ocean-Bottom-Hydrophones (OBH) were placed at 5 km intervals along the 245 km long profile. Except one station, OBH46, all receivers recorded shots from a seismic source consisting of 2 × 32-litres BOLT air guns, fired at 120 bars, providing a shot spacing of ~150 m.

**Figure 4:**
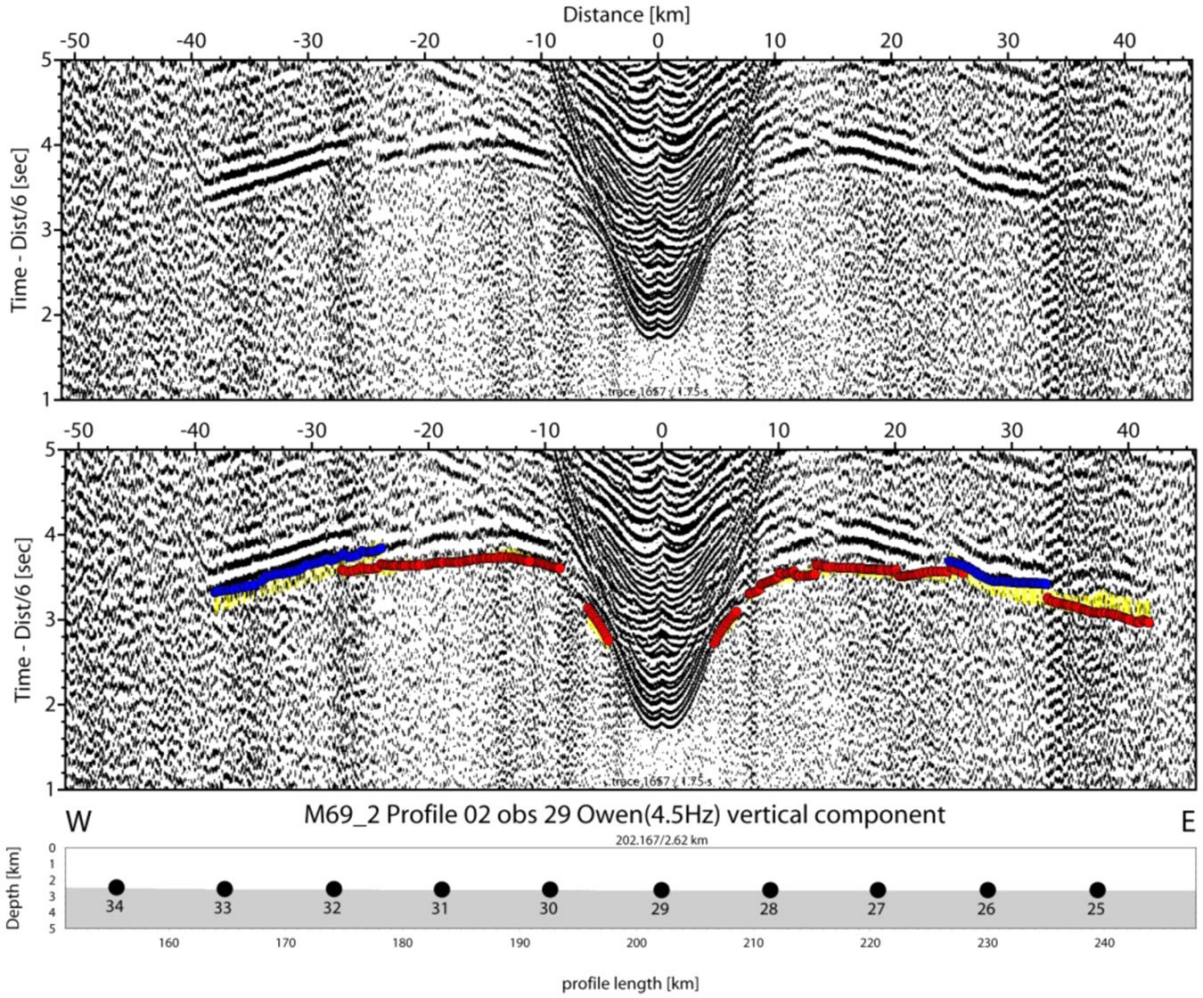
Seismometer recording of OBS29; yellow: picked arrivals with uncertainty; red: calculated crustal Pg arrivals; bule: calculated PmP reflection from crust/mantle boundary.

**Figure 5:**
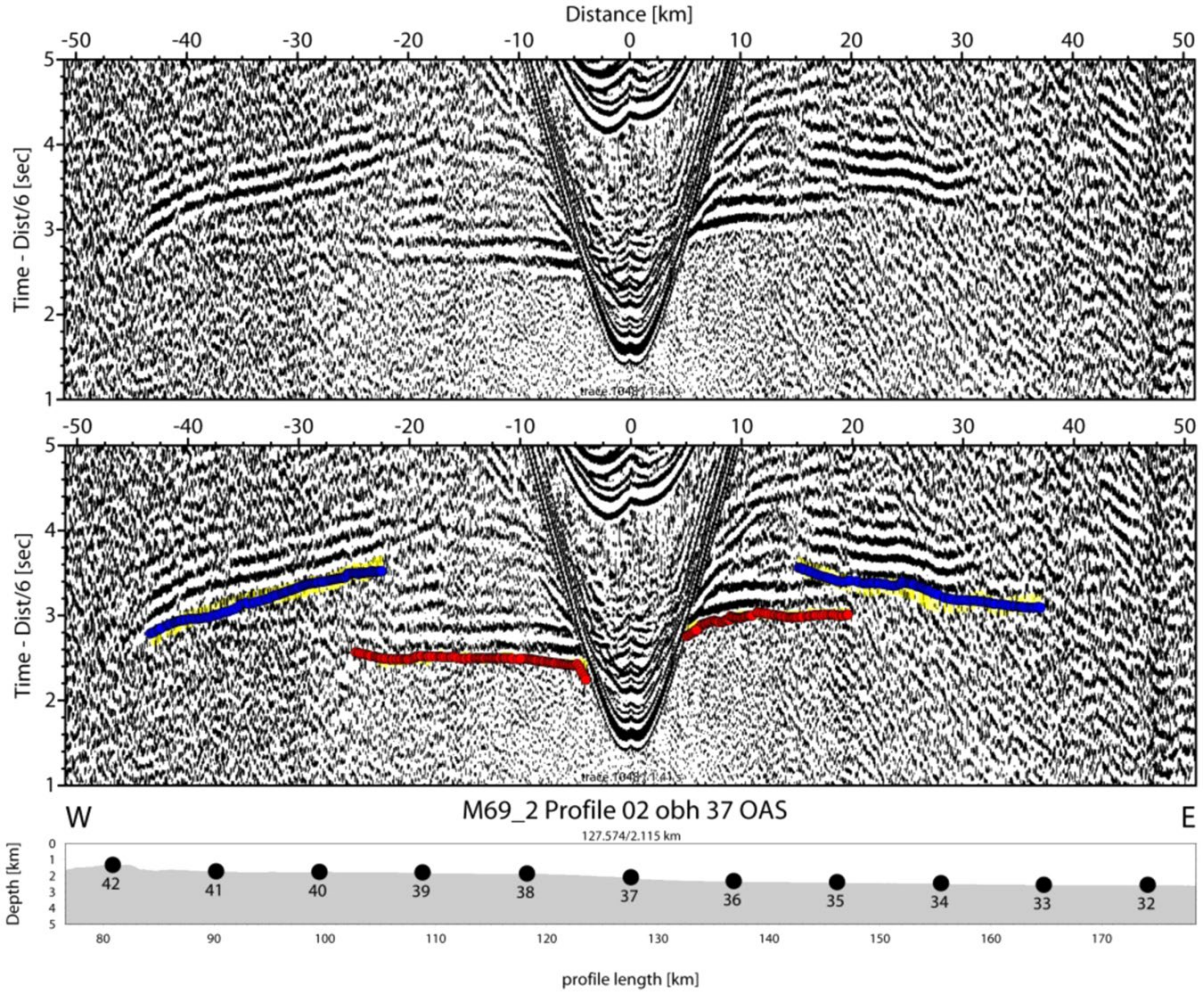
Hydrophone recording of OBH37; yellow: picked arrivals with uncertainty; red: calculated crustal Pg arrivals; blue (towards positive offsets): calculated PmP reflection from crust/mantle boundary; blue (towards negative offsets): calculated PiP reflection from inter-arc crust reflector.

**Figure 6:**
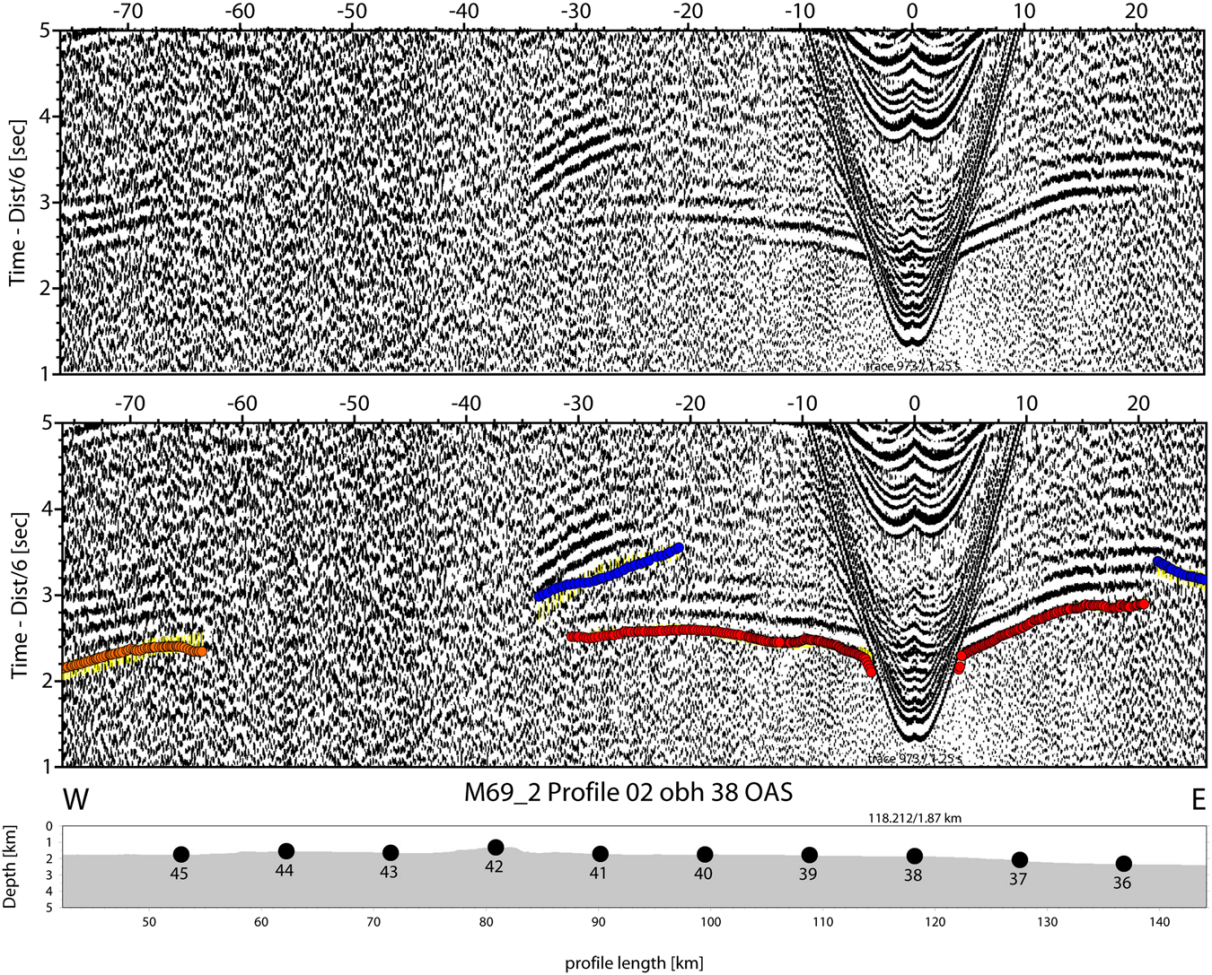
Hydrophone recording of OBH38; yellow: picked arrivals with uncertainty; red: calculated crustal Pg arrivals; blue (towards positive offsets): calculated PmP reflection from crust/mantle boundary; blue (towards negative offsets): calculated PiP reflection from inter-arc crust reflector; orange: reflection from the base of the arc crust.

**Figure 7:**
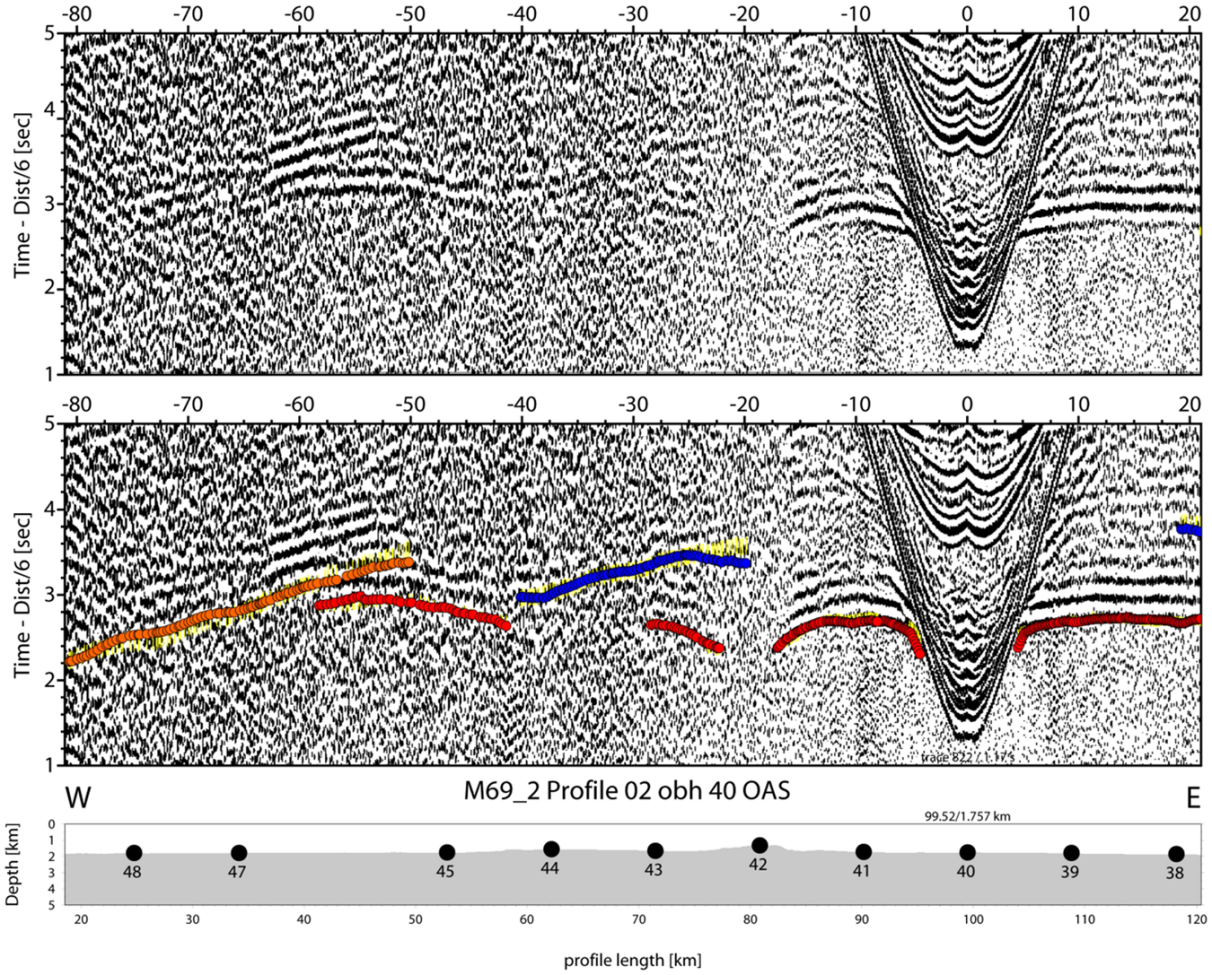
Hydrophone recording of OBH40; yellow: picked arrivals with uncertainty; red: calculated crustal Pg arrivals; blue: calculated PiP reflection from inter-arc crust reflector; orange: reflection from the base of the arc crust.

The Alborán Sea is known to be one of the busiest seas of the Earth, as basically all ship’s traffic entering the Mediterranean Sea through the Strait of Gibraltar runs through it. Profile P02 was co-located with the main traffic from Gibraltar towards the Suez channel. Therefore, seismic stations on the seabed suffer from a reasonably high-noise level. However, the waveforms of both refracted and reflected onsets show up nicely in most record sections and offsets of 40 to 60 km are characteristic for the majority of stations, for example OBS29 and OBH37 (Figs 4 and 5). The largest offsets of 70 to 90 km occur at OBH38 and OBH40 to the southeast of the Cabo de Gata (Figs 6 and 7). Sedimentary arrivals and most crustal arrivals could be picked to ±30–40 ms, or better. However, for larger offsets the decreasing signal-to-noise ratio causes larger uncertainties. The largest picking errors of ±90–100 ms have been assigned to secondary arrivals like wide-angle reflections and rays turning in the uppermost mantle.

Typical phases are a branch of arrivals turning in the sedimentary cover of 0.5 s TWT to 2 s TWT in thickness in seismic reflection data^25^ that is called Ps. The thickest sediments occur in the Algerian-Balearic basin. Here, seismic reflection data support Messinian evaporites and diapirs^25^ and seismic refraction branches show evidence of velocity inversion below the Messinian salt deposits (Fig. 4). Energy turning in the igneous crust, called Pg, occurs at offsets of ~8 km out to 25 km in the Algerian-Balearic basin (Figs 4 and 5). Father west, where the crust is thicker, Pg arrivals show up out to ~60 km (Fig. 7). In addition, most stations show a clear wide-angle reflection emerging at offsets on ~20 to 30 km. Stations being located in the Algerian-Balearic basin seem to sample the crust-mantle boundary, PmP. For branches providing wide-angle reflections westward of longitude 1.5°W, the phase seems to change in character and corresponds to an intra-crustal/arc reflection, called PiP. The PmP in the East Alborán Sea occurs at larger offsets of >60 km and is well sampled at a number of OBH, including OBH38 and OBH40 (Figs 6 and 7). In addition, OBS and OBH in the Algerian-Balearic basin sampled a seismic refraction branch from the upper mantle, Pn. Apparent velocities indicate upper mantle velocities of ~7.8 km/s.

### Seismic data inversion

Seismic refraction and reflection travel time data were inverted to obtain a crustal and upper mantle velocity model using the joint refraction and reflection travel time inversion method of Korenaga *et al*.^28^. This tomographic method allows determining a two-dimensional velocity field and the geometry of a floating reflector from the simultaneous inversion of first arrival and wide-angle reflection travel times. The velocity field is parameterized as a mesh of nodes hanging from the seafloor. The floating reflector is represented as an independent array of linear segments with only one degree of freedom (vertical) for each reflector node. We used the floating reflector to model the crust-mantle boundary, i.e., the Moho. The forward problem for both refracted and reflected phases is solved using a hybrid ray-tracing scheme based on the graph method^29^ with local raybending refinement^30^. Smoothing constraints using predefined correlation lengths and optional damping constraints for the model parameters are employed to regularize an iterative linearized inversion. A detailed description of this method is given elsewhere^31,32^.

**Figure 8:**
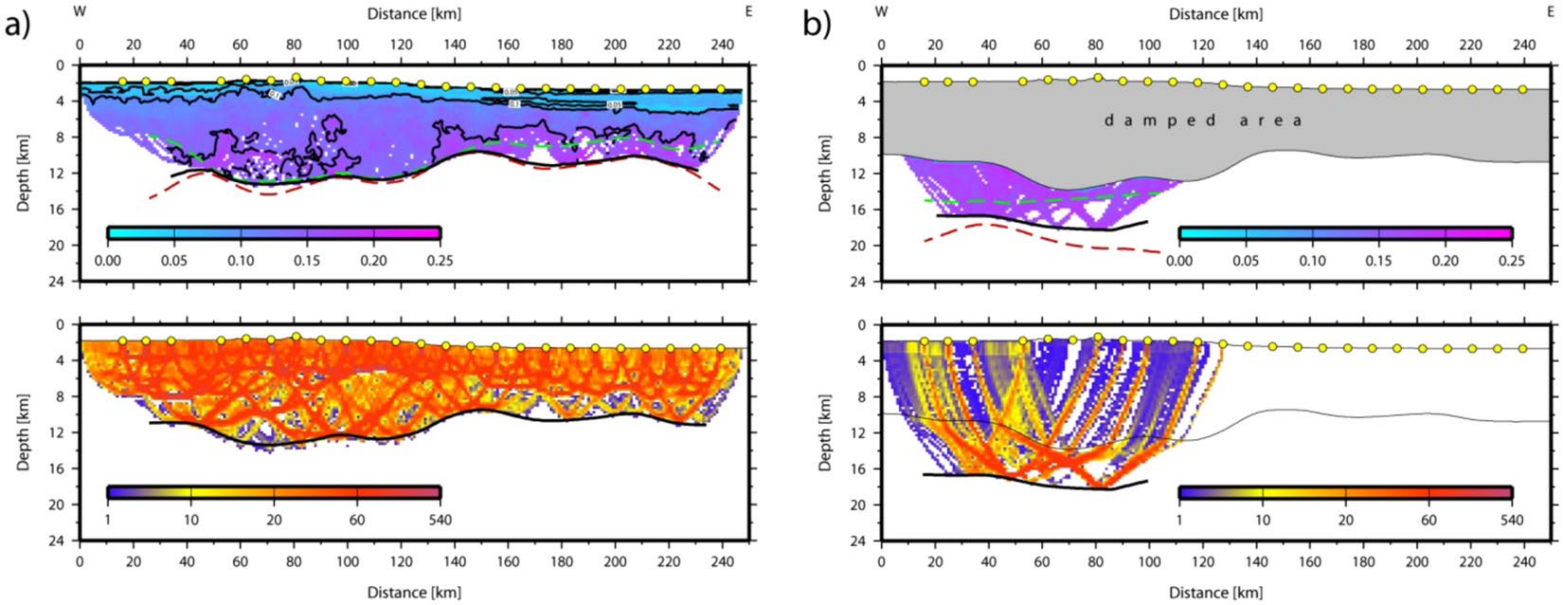
Model error/uncertainty (rms misfit (in seconds) obtained from 100 random input models and ray coverage of the average model); (**a**) upper part of the model, (**b**) volcanic-arc lower crust.

For the tomographic inversion we used a layer stripping method. Thus, the model was built up from top to bottom. The thickness and velocity structure of the sedimentary packages has been assessed and introduced in the initial model using the coincident MCS profile ESCI-Alb 2^25^ and Ps (n = 872) arrivals were inverted to refine the sedimentary velocity structure. Top of basement has also been obtained from the MCS data. Basement Pg (n = 5604) arrivals were inverted to obtain velocities of the igneous crust. Later, seismic reflections from intra-arc reflector and from the oceanic crust/mantle boundary (PiP + PmP, n = 2605) were added and inverted for both crustal velocity structure and depth to the floating reflector. This procedure resulted into an excellent fit of the data. The rms misfit was 61 ms and the χ^2^ < 1. To yield the lower-crustal structure of the Alborán volcanic arc, we added the crust/mantle boundary reflections (n = 872) observed to the west of OBH36 (see for example OBH38 and OBH40) and inverted for both velocity structure and thickness. Note, that the upper part of the model was damped to keep its velocity structure unchanged when inverting for the structure of the lower continental crust. Last, we added Pn arrivals (n = 214) to yield upper mantle velocities of the Algerian-Balearic Basin (Fig. 4). The robustness and errors of the final model were studied by using 100 different input models, varying the preferred model randomly by ±5%, indicating the errors tend to increase with increasing depth. However, errors are generally <0.2 km/s, providing a very robust final model with tight constraints on the velocity structure and crustal thickness (Fig. 8).

### Isostatic considerations on the volcanic arc topography

Thermal state controls lithospheric structure and with crustal structure and thickness is key to determine the isostatic state of a region. Further, isostasy may be influenced by dynamic forces related to the 3-D convection of density variations arising from geodynamic processes. In the case of the Gibraltar Arc – Alborán basin, the deep structure has been modelled to propose that changes in either slab-induced roll-back forces^5^ or alternatively slab-tear-induced^4,24^ lithospheric uplift caused the closure of the Rifean and Betic shallow marine corridors that linked Mediterranean and Atlantic water masses across north Africa and south Iberia, respectively. The closure of the marine corridors would have triggered the MSC and like previous works, these two models assume that some other undefined process had previously closed the Gibraltar Strait, but the biota speciation paths described above tell otherwise.

Here we aim at estimating quantitative constraints on the isostasy of a growing magmatic arc to further understand its role in the paleogeography of the region. For the sake of simplicity, we assume that the dynamics of the westward migration of the slab were reasonably steady state for the Late Miocene time, when the magmatic arc and back arc crust formed, although kinematic reconstructions for this time are poorly constrained. The magmatic arc is located next to 15–20-km-thick intruded continental crust, and at the time of magmatic growth the subducting Triassic-Jurassic slab must have been some 150–200 km deep under the magmatic arc and back arc. Thus, we assume that the typical average ~2.5 km depth of isostatically compensated ~6 km thick oceanic of active mid ocean ridges can be used as a reference depth, and comparatively estimate the expected depth of thicker magmatic arc crust.

**Figure 9:**
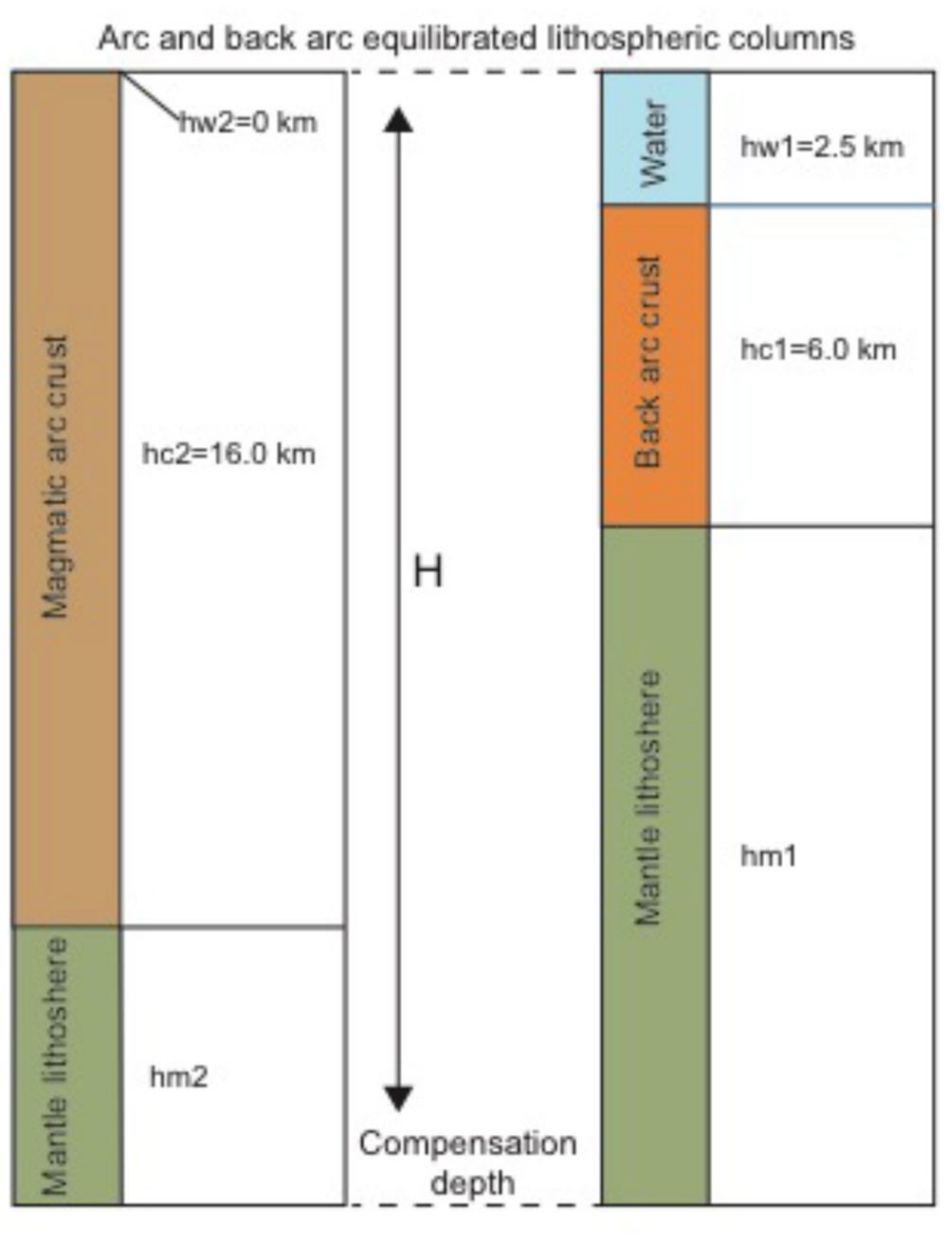
Sketch (not to scale) showing two isostatically balanced lithospheric columns. hw_1_ and hw_2_ are water depth, hc_1_ and hc_2_ are crustal thickness, and hm_1_ and hm_2_ are lithospheric-mantle thickness. A simple mass balance assuming an Airy model of isostasy predicts: ∆hw = 0.25 ∆hc^4^. Therefore, the top of a 16 km thick crust would reside 2.5 km shallower (left column) than the top of a 6 km thick crust (right column).

Thermal models currently seem to predict correctly the 2.5 km water depth at which crust reside at mid ocean ridges. However, thicker and thinner crust must reside at different depth solely from isostatic considerations. Consider two columns of lithosphere of the same thickness H isostatically compensated but with different crustal thickness (Fig. 9). A simple mass balance assuming an Airy model of isostasy predicts:

1 $${{\rm{\rho }}}_{{\rm{w}}}\,{{\rm{hw}}}_{1}+{{\rm{\rho }}}_{{\rm{c}}}\,{{\rm{hc}}}_{1}+{{\rm{\rho }}}_{{\rm{m}}}\,{{\rm{hm}}}_{1}={{\rm{\rho }}}_{{\rm{w}}}\,{{\rm{hw}}}_{2}+{{\rm{\rho }}}_{c}\,{{\rm{hc}}}_{2}+{{\rm{\rho }}}_{{\rm{m}}}\,{{\rm{hm}}}_{{\rm{2}}}$$

2 $${\rm{H}}={{\rm{hw}}}_{1}+{{\rm{hc}}}_{1}+{{\rm{hm}}}_{1}={{\rm{hw}}}_{2}+{{\rm{hc}}}_{2}+{{\rm{hm}}}_{2}$$

where ρ_w_ is water density, ρ_c_ is crustal density and ρ_m_ is mantle density and hw_1_ and hw_2_ are water thickness at columns 1 and 2 respectively, hc_1_ and hc_2_ are crustal thickness at columns 1 and 2 respectively, and hm_1_ and hm_2_ are lithospheric mantle thickness at columns 1 and 2, respectively. From combining expressions 1 and 2 we obtained:

3 $${{\rm{hw}}}_{1}\,\mbox{--}\,{{\rm{hw}}}_{2}=({\rm{\rho }}m\mbox{--}{\rm{\rho }}c)/({\rm{\rho }}m-{\rm{\rho }}w)\ast ({{\rm{hc}}}_{2}-{{\rm{hc}}}_{1})$$

Assuming average ρ_m_ of 3.33 g/cm^3^, ρ_c_ of 2.75 g/cm^3^ and ρ_w_ of 1.03 g/cm^3^ gives

4 $${\rm{\Delta }}\mathrm{hw}=0.25\,{\rm{\Delta }}\mathrm{hc}$$

Expression 4 indicates a 250 m water depth change for every 1 km of crust, or a 2.5 km shoaling of the topography of a 16 km thick basement compared to normal 6 km oceanic crust, effectively bringing the east Alborán magmatic arc to sea level. The Vp crustal structure mapped with wide-angle seismic data yields 6–7 km for the South Balearic – Algerian basin oceanic crust and 14–17 km thick crust for the volcanic arc area, which suggests that much of the arc was at sea level, in agreement with inferences made from the regionally mapped erosional Messinian unconformity at the crest of the magmatic structure^27^.

## Results

### Interpretation of Vp model

**Figure 10:**
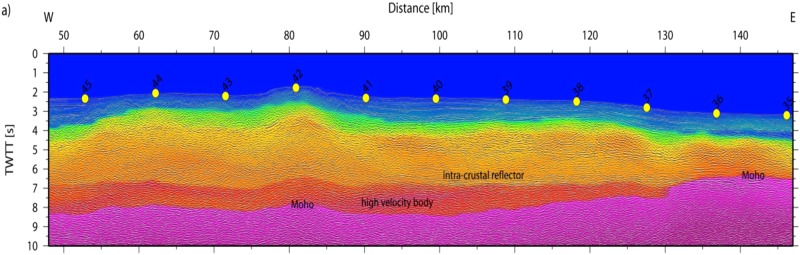
Algerian-Balearic Basin: multi-channel seismic (MCS) reflectivity plotted with the seismic velocity structure from the tomographic inversion; note, data are plotted as a function of two-way travel times (TWTT).

**Figure 11:**
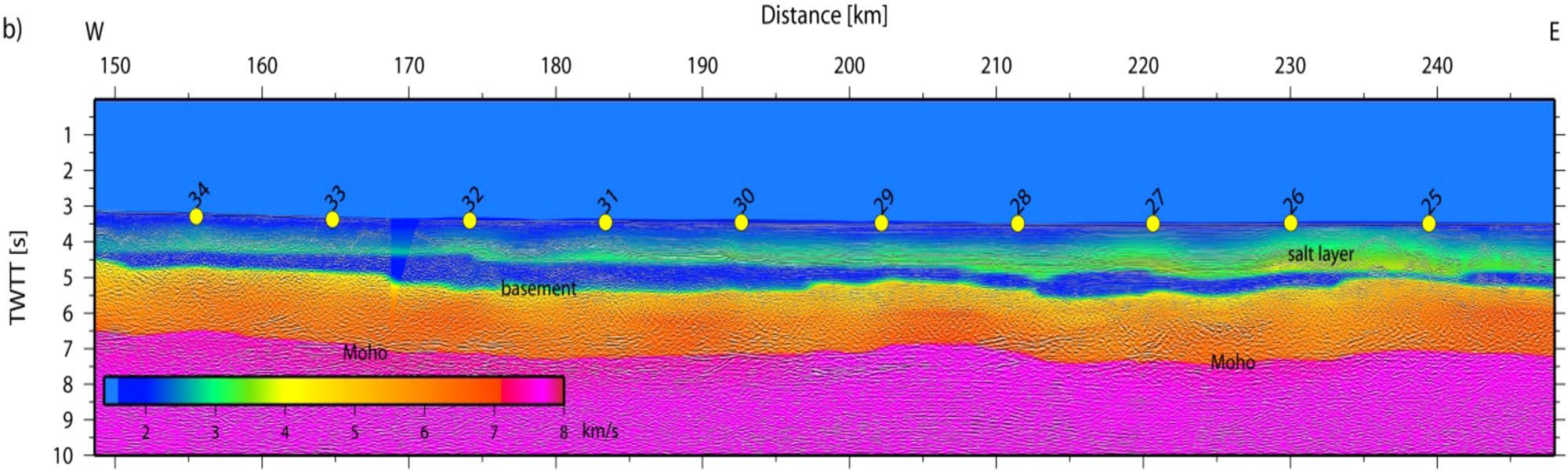
East Alborán Basin/volcanic arc: multi-channel seismic (MCS) reflectivity plotted with the seismic velocity structure from the tomographic inversion; note, data are plotted as a function of two-way travel times (TWTT).

The final model indicates two distinct domains and supports constraints derived from MCS data^25^ (Figs 10 and 11). In the east (Fig. 10), the igneous basement is covered by up to 3 km of sediment, including high-velocity Messinian salt deposits and a crust with a thickness of approximately 5 km. The high gradient in the upper crust and low gradient in the lower crust support oceanic-type crust formed by decompressional melting at a spreading center. Upper mantle velocity was in the order of ~7.8 km/s. Further west (approximately west of OBH36; Fig. 11) the seabed and basement rise from a nearly constant depth of ~2.5 km to ~1.8 km (near OBH42). The change in seafloor depth is associated with a change in the underlying crustal structure and an increase in crustal thickness (12 to 16 km), featuring the domain of the Alborán volcanic arc. A key feature of the wide-angle data is the high-velocity lower crust (Vp > 7.1–7.3 km/s), which is separated from the upper crust by a first order discontinuity, marked by a prominent PiP reflection in the wide-angle data and band of layered reflectors in the MCS data (Fig. 11).

### Messinian-Pliocene transition in the West Alborán Basin (WAB)

**Figure 12:**
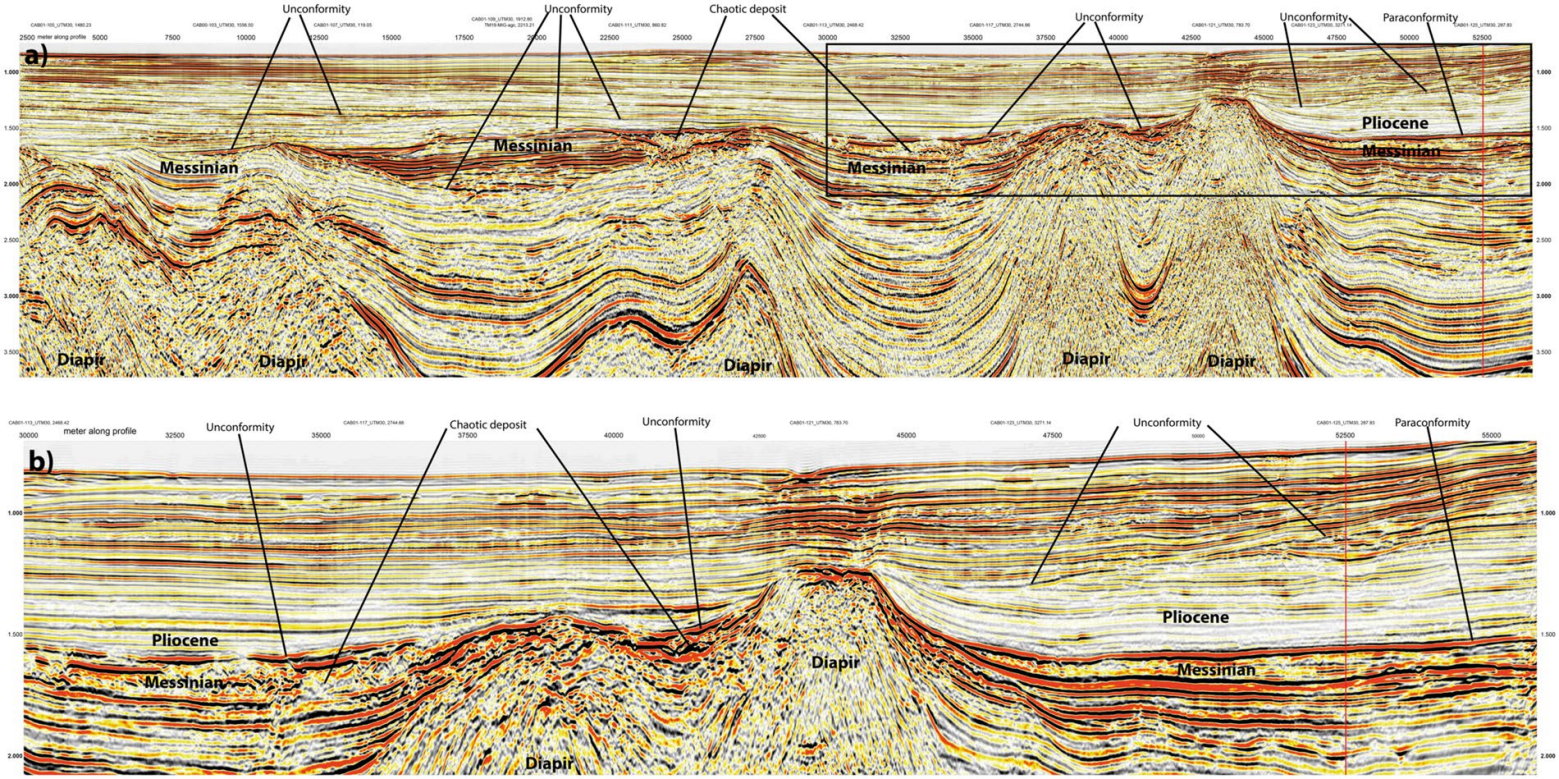
Seismic reflection line CAB01-104 from the WAB (See location of this line in Fig. 2). The line runs roughly parallel to the Iberian continental margin along the upper slope approaching the Gibraltar strait near its SW end. (**a**) The line shows a complex pattern of unconformities and laterally limited deposits that appear related to the compartmentalization created by the mud diapirs. (**b**) Close up of the SE end of the line marked by a black box in panel (a). Notice the intra-Pliocene unconformity that must have formed under submarine conditions, and thus, probably also other unconformities in this region. Notice that the contact between Messinian and Pliocene appears as a paraconformity in the NE end of the line. Red vertical line marks cross point with line CAB01-125 (Fig. 13).

**Figure 13:**
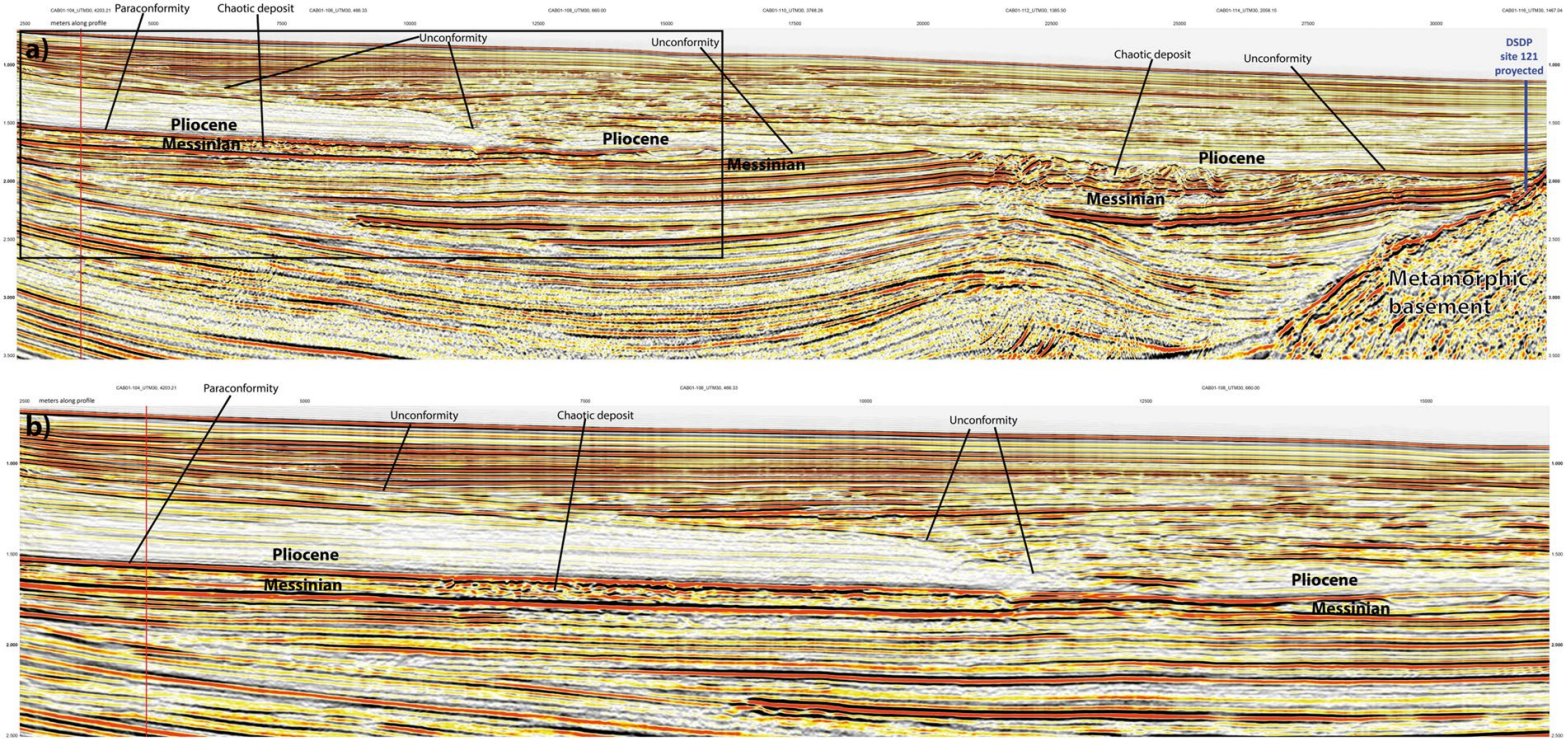
Seismic reflection line CAB01-125, perpendicular to the margin (See location in Fig. 2). (**a**) Notice Pliocene-Messinian erosional unconformity along much of the line that follows as a paraconformity under the upper slope. (**b**) Close up of the NW end of the line marked by a black box in panel (a). Notice the prominent intra-Pliocene erosional unconformity and the paraconformity of the Pliocene-Messinian contact in the uppermost slope. The paraconformity is underlain by somewhat chaotic deposits that grade into well-stratified reflections upslope. Red vertical line marks cross point with line CAB01-104 (Fig. 12).

For imaging the Messinian-Pliocene transition in the WAB we selected two industry reflection seismic lines, CAB01-104 and CAB01-125. These lines are located in the northern margin of the WAB where supposedly the MSC is represented by a subaerial unconformity at the base of the Zanclean, early Pliocene, deposits^8,21^. The Zanclean unit shows poor reflectivity and relatively continuous reflectors (Figs 12 and 13). This unit contrasts with the more reflective Messinian succession that is locally capped by a chaotic package of reflectors related to mass-transport processes and generally attributed to the MSC erosive period^8,21,33^ (Figs 12 and 13). The Zanclean unit is capped by an erosive unconformity that separates it from an overlying more reflective package of later Pliocene sediments. This intra-Pliocene unconformity cuts into the succession omitting great part of the Zanclean unit, locally reaching the Messinian, especially in structural-highs related to mud diapirs (Figs 12 and 13). An angular unconformity occurs between the Messinian and the Zanclean sediments around the flanks of mud diapirs, probably related to the activity of these structures (Fig. 12). However, laterally, in the region where lines CAB01-104 and CAB01-125 cross each other, the base of the Zanclean defines a paraconformity with the underlying more reflective Messinian deposits, which show parallel reflectors (Figs 12 and 13). Thus, one of the main erosive unconformities observed in the seismic lines has an intra-Pliocene age, dated at Site ODP hole 976^26^. The unconformity at the base of the Zanclean seems related to local tectonics associated to mud diapirism. Furthermore, in some regions of the WAB the Messinian-Pliocene transition is continuous with no evidence of erosion or evaporite deposits (Figs 12 and 13).

## Discussion

### The Alboran volcanic arc

**Figure 14:**
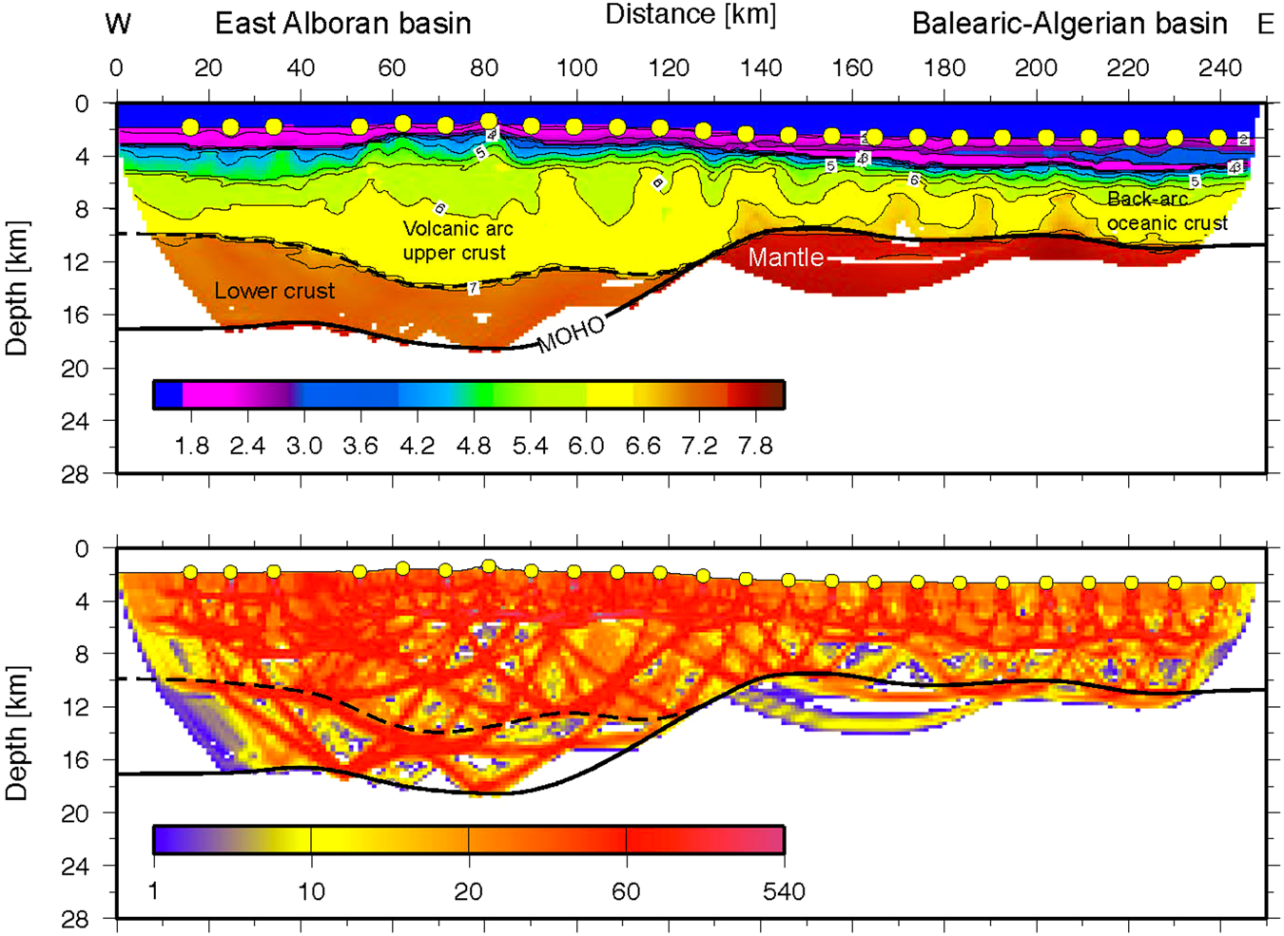
Seismic velocity (Vp) model of line P02 located across the Alborán Volcanic Arc and the Balearic-Algerian Basin to the East. The Vp model was obtained from tomographic inversion of travel time first arrivals and reflections (top). Ray coverage expressed as derivative weight sum (DWS) (bottom). Location of line in Fig. 1.

Line P02 provides the first detailed P-wave velocity (Vp) distribution of crust and uppermost mantle to constrain rock types in the Alborán basin (Fig. 14). The Vp model shows the change from an oceanic-type crust in the BAB to a different crustal structure in the EAB with features previously unknown. The crustal structure changes from the BAB to the EAB in thickness, velocity-depth distribution, and, particularly in lower-crust velocity. The BAB displays a ~7 km-thick basement with a Vp-depth distribution typical of the layers 2/3 of an oceanic-crust velocity structure, albeit here lower-crust velocities typically are <7 km/s, somewhat lower than Vp of crust formed at mid-ocean ridges^34^. In contrast, the EAB has a 16–17 km-thick basement, with a 2–4 km thick high-Vp lower-crustal layer that indicates a different composition (Fig. 14). This layer of Vp > 7.2 km/s is unusual compared to velocities of lower crust in Iberia and North Africa^35^ or continental lithologies exposed in the Betics–Rif. Continental crust normally lacks a high-velocity lower crust^36^. Two plausible explanations for the high velocities are serpentinized mantle, similar to the onshore Ronda peridotites^37^ or layered mafic cumulates similar to those exposed in exhumed arc sections^38,39^. Although forearc mantle serpentinization by fluids from dehydrating slabs has been interpreted in subduction zones, this occurs in the cold forearc and not at the hotter mantle wedge with volcanic arc-type activity at its surface. Further, the base of the high-velocity lower crust is defined by wide-angle continuous reflections, which is unusual in the case of the transition typical of serpentinization.

A high-velocity lower crust is a distinct feature of a number of volcanic arcs, including Tonga^32^, Izu-Bonin^40^ and Aleutians^41,42^. Moreover, crustal thickness, velocity-depth distribution, and especially this high-Vp lower crust, compares well to the structure of Pacific volcanic arcs^40,42^. While the transition to the lower crust might be more gradual at the Tonga arc^32^ and Izu-Bonin arc^40^, it is separated from the upper crust by a distinct first order discontinuity in the Aleutians^42^, mimicking features found in our data.

Basement sampling near line P02 found tholeiite arc-type rocks with no continental crust geochemical signature^4,5^, supporting that the high-Vp lower crust is related to arc mafic cumulates, rather than cold-mantle serpentinization. Mafic cumulates in the Alboran volcanic-arc lower crust would be devoid of garnet due to the shallow crustal thickness and possibly similar to those exposed in the Talkeetna arc in Alaska, comprising layered piroxenites and gabronorites^39^. Thus, the Vp model supports the existence of a magmatic arc in the EAB.

### The East Alboran volcanic archipelago

The formation of a ~17 km-thick arc crust underlain by an active mantle wedge in a subduction-related geodynamic setting has implications for the paleo-geographic evolution of the region.

Although the thermal and density structure of the mantle below the arc and the role of dynamic forces are difficult to establish, basic isostatic considerations shown above indicate that the top of the magmatic 16–17 km thick crust must have been at about sea level forming an archipelago with shallow sills across the EAB. Conceivably, several processes that may locally influence isostasy are not included in our first-order estimation. It has been shown that pulses in Reykjanes hotspot magmatic activity moving along a spreading center cause regional uplift that leaves a record in sedimentary sequences^43^. Similarly, magmatic intrusions in the continental crust have an effect in short-term crustal isostatic alterations by first producing punctuated uplift followed by smaller subsidence produced during magma cooling, which is typically faster than thermal subsidence^44^. Given that the magmatic arc developed for several millions of years, it is likely that time-variability in magmatic activity has had a waning and waxing effect in arc relief and may have paid a role in the location of channels regulating water replenishment into the Mediterranean during the MSC, needed to produce the large volume of halite salt in the evaporites, imaged on seismic data in the deep basins. However, details of the arc structure, made entirely of magmatic products from flux melting and perhaps some decompression melting, and the temporal variability of the construction processes are currently unconstrained.

In summary, the buoyancy of a 14–17 km thick arc crust brings the top of the arc close to sea level, and magmatic pulses during construction and tectonic processes during arc migration and collision may cause temporal changes in the regional configuration of the arc relief.

As the subduction zone migrated westwards^25^, the volcanic archipelago developed in the EAB forming the Cabo de Gata region, with fieldwork indicating gradual emergence since 10 Ma^41^. We propose that the volcanic region extended to the south to reach the African continental platform and the coastal zone of the eastern Rif, where similar volcanic arc rocks crop out^4,5^ (Fig. 2b). Several independent observations arising from seismic stratigraphy analysis of the evolution of the EAB support this hypothesis: (1) Mapping of late Neogene erosional unconformities supports that the currently submerged volcanic arc had emerged regions across the EAB^27^. To the east and west of the arc there is evidence of Messinian age deposits that are lacking over most of the volcanic arc domain. (2) Pliocene to Quaternary strata progressively lap on the volcanic basement^25,27^, indicating that the arc submerged progressively after volcanism ceased in Messinian time, with large islands persisting until the Pleistocene^27^.

### Tectonic mechanisms influencing the volcanic arc topography

Notwithstanding the above isostatic considerations that support a Late Miocene emerged volcanic arc, other geodynamic mechanisms probably also concurred, influencing the topography of the archipelago during the late Miocene and later. The narrow structure of the Gibraltar Arc – Alborán basin system is underlain beneath Iberia and NW Africa by a steep slab, wich is partially detached under east Iberia. The slab extends at depth to the 660 km discontinuity. The slab has not been detected by geophysical methods and possibly does not exist under the SE Alborán basin and corresponding African continental margin^45^. Thus, the 3D density structure of the system is complex and possibly plays a considerable role in the distribution of large-scale regional topography particularly in sectors above the slab.

Slab rollback and associated isostatic rebound may have played an additional role in the topography of the volcanic arc and the WAB. Since 9–10 Ma the slab underlying the Alboran basin has retreated westwards approximately 200 km, from being bellow the active volcanic arc around meridian 2°W between 8–10 Ma^5^, to presently producing intermediate seismicity at depths of 100 km at meridian 4.5°W^46^. Onshore Late Messinian to Pliocene marine deposits registered a pulse of subsidence followed by uplift that migrated from east to west along the Iberian and Moroccan coast^47^ that may reflect this westward slab rollback. A relationship between Mediterranean desiccation during the MSC and an increase in volcanism due to decompression melting has been suggested for the western Mediterranean^48^, however, in the studied region, most of the Messinian magmatism both onshore and offshore occurred before the Messinian salinity crisis, except in the southwestern end of the volcanic arc onshore at the Rif^4^.

### Faunal-exchange and geological implications of the Alboran volcanic arc

The Alboran volcanic archipelago was located in the region that best explains the terrestrial fauna speciation patterns resulting from taxa exchange between North Africa and Iberia (Figs 1 and 2). It solves the requirement for an emerged land biological hotspot during the Tortonian (10–7 Ma) located in the South Eastern Betics^13^. Furthermore, the existence of an archipelago between SE Iberia and the eastern Rif would explain the exchange of terrestrial taxa before the MSC^12–16^, acting as a filter bridge for aquatic mammals like *Hippopotamus* before the main terrestrial faunal exchange at 6.2 Ma^17–19^. Thus, the observed terrestrial biota diversification paths and large vertebrate fossil record support a land-bridge linking SE Iberia to the Eastern Rif, far east of the Gibraltar Strait through the Alboran volcanic arc (Figs 1 and 2).

Further, five independent geological observations support a Messinian-Pliocene land-bridge in the EAB rather than at Gibraltar, and a sustained connection of the WAB with Atlantic water masses during the MSC. (1) The Messinian-Pliocene pelagic sequence cored at ODP site 976 in the WAB found no evidence of a change in water depth. This borehole was located at the edge of a basement high and great part or all of the upper Messinian was eroded during the Zanclean, but no change in sedimentary facies is observed across the Messinian-Pliocene transition^26^ (Fig. 1). (2) The Mediterranean basin repopulation in the Pliocene by characteristic Mediterranean pre-MSC fauna requires an open-marine refuge that preserved biota during the MSC^49^. (3) Upper Messinian lago-mare sediments cropping out onshore at the northern margin of the WAB include Atlantic marine biota^50^. (4) MSC sediment units in deep Mediterranean basins contain salt deposits easily detected with seismic records e.g.^25^. However, no salt deposit is imaged in the WAB, which was 1.5–2 km deep at the onset of the MSC^26^. The scenario of a growing Tortonian-Messinian arc that formed a sill in the EAB may well explain the puzzling contrast in the distribution of Messinian salt deposits. Salt is absent in the depocenter of the WAB, but is hundreds of m-thick east of the EAB^25^. Thick salt deposits extend across the BAB floored by 6–7 km thick back-arc oceanic crust (Figs 2, 11 and 14) to pinch out to the west in the region of transitional crust (8–10 km) to the thicker crust (~16 km) of volcanic-arc type rocks (Figs 10 and 14). (5) Across Mediterranean basins, shallow regions contain an erosional unconformity related to the MSC sea level drop^1–3^. However, in the WAB the most pronounced channels near the Gibraltar strait may be older than Zanclean and probably formed under marine conditions^9^. Furthermore, the region of the uppermost slope across part of the WAB shows a complex pattern of erosive unconformities partly related to mud diapirs activity with Zanclean and intra Pliocene age (Figs 11 and 12). Finally, and more important, laterally along the uppermost slope of the WAB the Pliocene-Messinian boundary is a paraconformity, both in North Africa^33,51^ and in Southern Iberia, with no evidence of subaerial erosion (Figs 11 and 12).

## Conclusions

We propose that ~10–6 Ma magmatism created a land bridge, possibly emerging most of the time as an archipelago extending from southeast Iberia to the eastern Rif, which determined the paths of speciation and the choking of the Mediterranean, modulating the MSC. During the MSC the Alboran volcanic arc separated an open marine WAB realm from the restricted Mediterranean to the east of the arc. First, the archipelago during the early stages of volcanic arc development (6.2–10 Ma) permitted the faunal differentiation observed in the eastern Betics^13^ and worked as a filter bridge for certain species, and later the more evolved volcanic land-bridge (6.2–5.3 Ma) permitted taxa exchange between the southeastern region of Iberia and the eastern Rif (Fig. 1). After arc magmatism waned, cooling of the crust and underlying mantle thermal boundary caused lithospheric thickening and thermal subsidence ending the MSC, and gradually limiting the faunal exchange across the archipelago, although large islands remained until the Early Pleistocene.
